# LncRNA FTX activates FOXA2 expression to inhibit non–small‐cell lung cancer proliferation and metastasis

**DOI:** 10.1111/jcmm.15163

**Published:** 2020-03-16

**Authors:** Shidai Jin, Jing He, Yue Zhou, Deqin Wu, Jun Li, Wen Gao

**Affiliations:** ^1^ Department of Oncology The First Affiliated Hospital of Nanjing Medical University Nanjing China; ^2^ Department of Thoracic Surgery The First Affiliated Hospital of Nanjing Medical University Nanjing China; ^3^ Department of Pharmacy The First Affiliated Hospital of Nanjing Medical University Nanjing China

**Keywords:** FOXA2, FTX, miR‐200a‐3p, non–small‐cell lung cancer

## Abstract

Lung cancer leads to the highest mortality among all cancer types in the world, and non–small‐cell lung cancer (NSCLC) occupies over 80% of the lung cancer cases. Numerous studies have demonstrated that long non‐coding RNA (lncRNA) is involved in various human diseases including cancer. LncRNA FTX was firstly identified in Xist gene locus and was dysregulated in many human cancers. However, the function of FTX in NSCLC is still unclear. Here, we report that long non‐coding RNA FTX expression level is down‐regulated in NSCLC clinical tissue samples and cell lines. Ectopic expression of FTX inhibits proliferation and metastasis of lung cancer cells in vitro and in vivo. Furthermore, we find that FTX overexpression activates the expression of transcription factor FOXA2, an important regulator in lung cancer progression, and we reveal a novel FTX/miR‐200a‐3p/FOXA2 competing endogenous RNA regulatory axis in lung cancer cells. Our results provide new insights and directions for exploring the function of FTX in lung cancer progression.

## 1 INTRODUCTION

Lung cancer is one of the most common cancers in the world and leads to the highest mortality in the past decades.^1^ NSCLC accounts for more than 80% of all lung cancer patients and is further classified by pathological characters into adenocarcinoma, squamous carcinoma and large cell carcinoma.^2^ Currently, surgery, chemotherapy and radiotherapy still occupy the most portion of NSCLC treatment.^3^ Although patients at stage I, II or IIIA develop resectable tumours that can be removed through surgeries, tumours may still relapse and even invade to other tissues within months.^4^ Moreover, difficulties in early diagnosis of NSCLC contribute to higher mortality and poorer prognosis. More than 60% of NSCLC patients were found to have advanced‐stage diseases at first diagnosis (stage III or IV).^5^ Thereby, understanding of molecular mechanism and identification of prognostic markers of NSCLC will help us to evaluate clinical trials for NSCLC patients and lead to take further steps.

Whole‐genome sequencing suggests that protein coding genes only occupy 1.5% of the genome, while the rest non‐coding genes have been proved to play vital roles in complex cellular processes nowadays. Long non‐coding RNAs (lncRNAs) are non‐protein coding nucleotides with length longer than 200nt and distinguished with other small non‐coding RNAs like siRNA, microRNA and piwiRNA.^6^ LncRNA FTX (five prime to Xist) was firstly identified by comparative sequence analysis around Xist gene locus and found to be evolutionarily conserved in mouse and human.^7^ In the first place, FTX was demonstrated to be up‐regulated during female embryonic stem cell differentiation and be able to activate Xist gene expression which involves in X chromosome inactivation.^8^ More studies then imply that it also relates to other biological processes during tumorigenesis and cancer development. Liu et al showed that FTX could compete with miR‐374a to inhibit epithelial‐mesenchymal transition (EMT) of hepatocellular carcinoma (HCC) cells or inhibit their proliferation by binding to an E3 ligase MDM2.^9^ Another study reported that FTX could activate ALG3 expression by eliminating interference of miR‐342 in acute myeloid leukaemia (AML).^10^ However, whether lncRNA FTX participates in lung cancer progression and the possibility for FTX to act as a prognostic marker remain unclear.

In our study, we found that FTX expression was decreased in NSCLC clinical tissue samples and cell lines. FTX overexpression inhibited lung cancer cell proliferation and metastasis via interacting with miR‐200a‐3p to promote FOXA2 expression, suggesting that FTX may act as a tumour suppressor in lung cancer progression.

## 2 MATERIALS AND METHODS

### 2.1 Tissue samples

Biopsy samples of NSCLC tissues and paired normal tissues were collected from patients undergoing surgeries at The First Affiliated Hospital of Nanjing Medical University. All the patients were aware of the experimental procedures and provided informed consent. Aspects of this research were approved by the Ethics Committee from The First Affiliated Hospital of Nanjing Medical University.

### 2.2 Cell culture

A549, H23, HCC827, H1299 and IMR90 cells were purchased from ATCC. Culturing media DMEM, RPMI1640 or MEM (Thermo Fisher Scientific) supplied with 10% (v/v) FBS (Invitrogen), and antibiotics (Thermo Fisher Scientific) were used to culture indicated cell lines at 37°C in an incubator containing 5% carbon dioxide with a humid environment.

### 2.3 Cloning vector construction, viral infection and cell transfection

For overexpression, FTX cDNA fragment was inserted into the XbaI/BamHI sites in pLVX‐IRES‐mCherry lentiviral expression vector (Takara Bio Inc). The cloning primers for FTX overexpression were as follows: forward primer 5’‐TCTAGAGGAAGCACACTGCGGCGATTC, reverse primer 5’‐GGATCCGGGAAATAAGTTTATTACATA. FTX overexpression vectors or control vectors were transfected together with lentiviral packaging system into 293T cells. Viruses were harvested after 48 hours and infected A549 and H1299 cell lines referring to previous protocol.^11^

Wild‐type and mutant FTX gene or FOXA2 3’‐UTR sequences were individually synthesized by GenePharm and cloned into pGL3‐basic luciferase reporter vector (Promega) for the following luciferase assay. The miR‐200a‐3p mimics and control miRNA mimics were synthesized by GenePharm. A549 and H1299 cells were transfected with pGL3 vectors or miRNA mimics using Lipofectamine 2000 following the manufacturer's instructions (Invitrogen).

### 2.4 Cell proliferation assay, EdU assay, cell apoptosis and cell cycle analysis

Cell growth of lung cancer cells was examined by the Cell Counting Kit‐8 (CCK‐8, Dojindo) and EdU Cell Proliferation Kit (Ribobio) following the manufacturers’ instructions. Cell apoptosis was examined using the Apoptosis Detection Kit (KeyGEN BioTECH). Cell cycle was assessed using the Cell Cycle Detection Kit (KeyGEN BioTECH).

### 2.5 Cell migration and invasion assays

For cell migration assay, transwell 24‐well permeable supports (8.0 µm pore) were used. Briefly, 1 × 10^5^ cells in DMEM were seeded onto upper membrane and DMEM with 10% FBS were provided to the bottom chambers. After 12‐hour incubation, cells that did not transfer through the polycarbonate filter were slightly removed. Migrated cells were fixed by 100% methanol and stained with 0.4% crystal violet. Images were captured, and cell numbers were counted under an inverted microscope (Nikon).

For cell invasion assay, transwell chambers supplemented with collagen‐coated membrane inserts (Corning) were used followed by same steps performed in migrated transwell assay.

For wound healing assay, indicated cells were plated in 6‐well plates and incubated overnight until confluence. Cell monolayers were scratched using 20‐µl pipette tips. After culturing for another 18 hours, photographs of the annealing wounds were taken using an inverted microscope.

### 2.6 Immunofluorescence (IF) assay

IF assays were performed in accordance with a standard protocol. Briefly, cells seeded onto a removable 8‐well chamber (ibidi) were fixed in 4% paraformaldehyde, incubated in PBS with 0.1% Triton X‐100 for 30 minutes and then blocked with 3% BSA in PBST for 30 minutes. Cells were incubated with diluted E‐cadherin primary antibody for 1 hour at room temperature. After washing with PBS for three times, cells were then incubated with appropriate Alexa Fluor‐conjugated secondary antibody (Thermo Fisher Scientific) for 1 hour in the dark. Staining using the DNA stain DAPI followed and images were taken and analysed by a confocal scanning microscopy (FV10i, Olympus).

### 2.7 Western blot analysis

Cells were collected and lysed using RIPA lysis buffer for following Western blot analysis. Briefly, cellular protein samples were loaded into a SDS‐PAGE at an even level. Then, proteins were transferred onto PVDF membranes using Trans‐Blot SD semi‐dry transfer cell (Bio‐Rad). After blocking with 5% skim milk for 1h at room temperature, the membranes were probed with indicated primary antibodies at 4°C overnight. PBST was used to wash the membrane for 4 times the second day. Then, the membranes were incubated with secondary antibodies at room temperature for 1h. After washing by PBST for 4 times, enhanced chemiluminescence substrates were used to detect protein bands in a dark room. The following antibodies were used for Western blots and the above IF assay: E‐cadherin (Cell Signaling Technology), FOXA2 (Abcam) and GAPDH (Abcam).

### 2.8 RNA isolation and quantitative RT‐PCR (qRT‐PCR)

Total RNA from frozen tissue samples or cell lines was isolated using TRIzol reagent according to manufacturers’ instructions. HiScript 1st Strand cDNA Synthesis kit (Invitrogen) was used to synthesize cDNA from prepared RNA samples. Real‐time PCR was performed using FastStart Universal SYBR Green Master mix (Roche) on ABI 7900HT Fast Real‐Time PCR system (Applied Biosystems).

### 2.9 Animal model and relative animal studies

All animal experiments were performed following the National Institutes of Health Guide for the Care and Use of Laboratory Animals. Experimental procedures were approved by Institutional Review Board of The First Affiliated Hospital of Nanjing Medical University.

BALB/C female nude mice (4 to 6 weeks old) and female SCID mice (4 to 6 weeks old) were obtained from the Model Animal Research Center of Nanjing University. For in vivo cancer cell growth assay, vector control or FTX‐OE A549 cells (5 × 10^6^) suspended in PBS/Matrigel (9:1) were subcutaneously injected into different flanks of nude mice (5 mice/group). Tumour volumes were monitored every 4 days. All animals were killed 4 weeks after injection, and excised tumours were weighed and prepared for following IHC analysis.

For in vivo cancer cell metastasis assay, SCID mice were separated into two groups (n = 5). Vector control or FTX overexpressed A549 cells (1 × 10^6^) resuspended in PBS were injected into mice through tail vein. Tumour growth was assessed using bioluminescence imaging (BLI) system (Caliper IVIS Lumina XR) every week. After a 30‐day breeding, mice were killed and lungs were harvested for H&E staining. Numbers of the lung nodules were observed and quantified microscopically.

### 2.10 Dual‐luciferase reporter assay

Previously described pGL3 reporter vectors were used for luciferase assay. Wild‐type or mutant vectors containing FTX gene or FOXA2 3’‐UTR region were transfected into A549 cells using Lipofectamine 2000, together with miRNA mimics and β‐gal plasmid which was considered to be measurement of transfection efficiency. After culturing for 48 hours, relative luciferase activities of cells were analysed using Dual‐Luciferase Reporter Assay Kit (Promega).

### 2.11 RNA fluorescence in situ hybridization (FISH) assay

FTX probes were synthesized by Ribobio, and the experiments were performed following the manufacturer's instructions of Fluorescent In Situ Hybridization Kit (Ribobio). Briefly, A549 and H1299 cells were fixed in 4% paraformaldehyde for 15 minutes and washed with PBS for three times. Then, cells were permeabilized using PBS containing 0.2% Triton X‐100. After PBS washing, cells were incubated in pre‐hybridization buffer at 37°C for 30 minutes. Then, cells were incubated in hybridization buffer containing FTX probe mix at 37°C overnight. After washing steps, cells were stained with DAPI for 10 minutes and viewed using microscope (Nikon).

### 2.12 RNA pull‐down assay

RNA pull‐down assay was performed following the instructions of RNA Pull‐Down Kit (Pierce). Briefly, FTX and FTX antisense (used as negative control) were cloned into pcNDA 3.1 vector and then in vitro transcribed using T7 RNA polymerase (Promega). These RNAs were labelled by biotin using RNA 3´ Desthiobiotinylation Kit and then incubated with Streptavidin Magnetic Beads. Then, these beads were mixed with cell extracts at 4°C overnight. After washing, the coprecipitated RNAs were extracted using TRIzol and measured by quantitative RT‐PCR.

### 2.13 Statistical analysis

All numeric data are represented as the mean ± SD of three independent experiments. Data statistical significance was calculated by Student's t* test*. Significance was defined as *P < .05 and **P < .01.

## 3 RESULTS

### 3.1 FTX expression is down‐regulated in lung cancer

**Figure 1:**
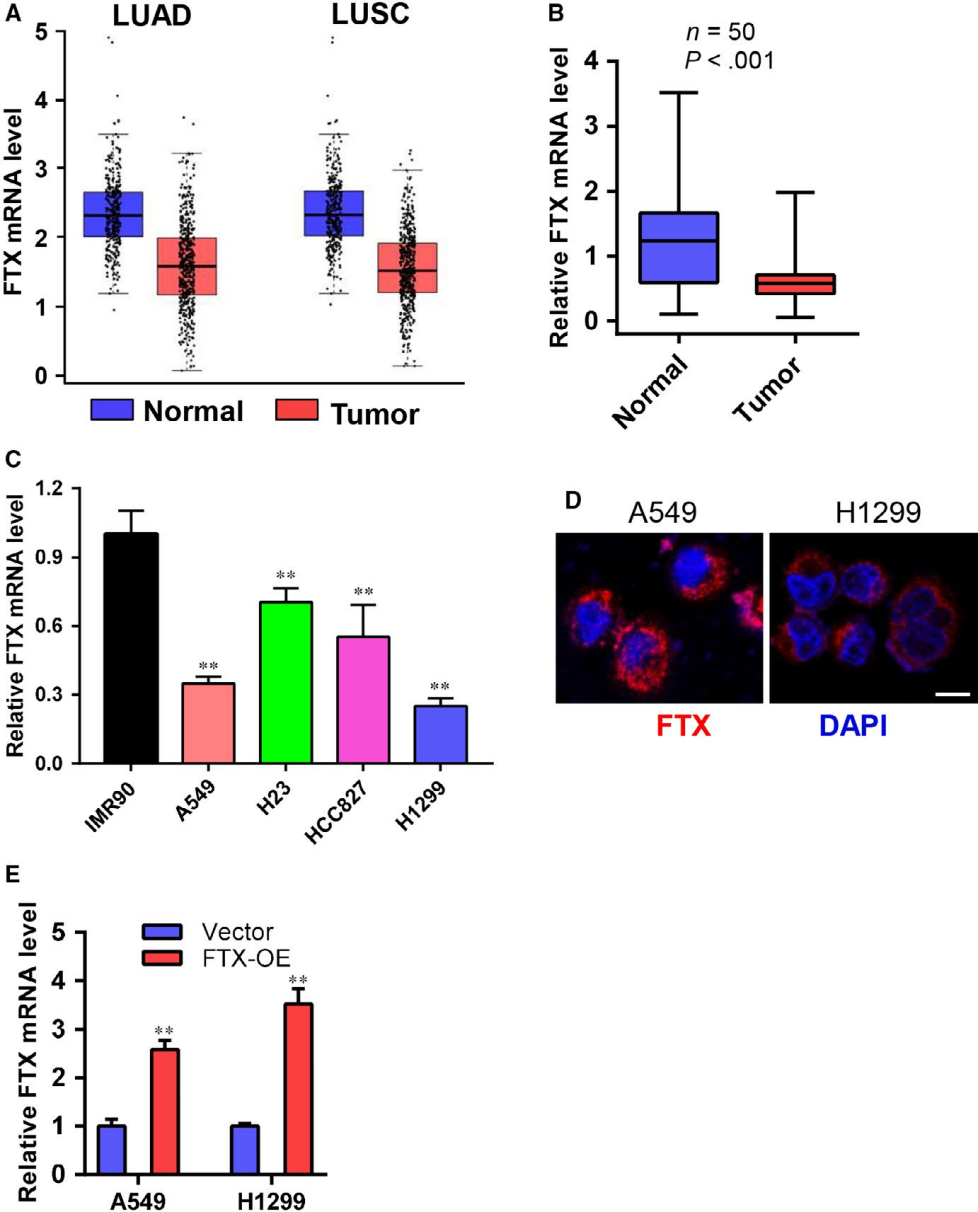
FTX expression is down‐regulated in lung cancer. A, FTX mRNA levels in lung adenocarcinoma (LUAD) and squamous carcinoma (LUSC) tissues. Data were obtained from TCGA database. B, Real‐time PCR analysis of FTX mRNA levels in collected NSCLC tissues and normal tissues (n = 50). *P* < .001 compared with normal tissues. C, Real‐time PCR analysis of FTX mRNA levels in IMR90, A549, H23, HCC827 and H1299 cells normalized to GAPDH. **P < .01 compared with IMR90 cells. D, Cellular distribution of FTX in A549 and H1299 cells was visualized by RNA‐FISH assay. Nuclei were stained by DAPI (blue). Scale bar 20μm. E, Real‐time PCR analysis of FTX mRNA levels in vector and FTX‐OE A549 and H1299 cells normalized to GAPDH. Values were shown as mean ± SD from three independent experiments. **P < .01 compared with control cells

To investigate whether the expression level of FTX had a significant change in NSCLC clinical samples, we first examined FTX expression profile in The Cancer Genome Altas (TCGA) database. We found that FTX mRNA levels were down‐regulated in lung cancer clinical tissue samples (Figure 1A). Then, we checked FTX mRNA levels in collected clinical samples from NSCLC patients, and real‐time PCR data suggested that 74% (37/50) of NSCLC tumour samples exhibited reduced FTX expression compared to adjacent normal tissues (Figure 1B). Furthermore, we analysed mRNA expression level of FTX in four independent lung cancer cell lines as well as in IMR90 lung fibroblast cells. The result indicated that FTX expression was decreased in lung cancer cell lines compared to IMR90 cells (Figure 1C). These data suggest that FTX expression levels are down‐regulated in lung cancer, and FTX may inhibit lung cancer malignant progression. Since there are no reports of FTX in lung cancer, we first decided to examine the subcellular distribution of FTX in lung cancer cells. The result of fluorescence in situ hybridization (FISH) assay indicated that FTX was mainly distributed in the cytoplasm (Figure 1D). To further explore the impact of FTX during lung cancer development, we overexpressed FTX (FTX‐OE) in A549 and H1299 cells using lentivirus. Real‐time PCR analysis confirmed ectopic expression of FTX gene in these cells (Figure 1E).

### 3.2 FTX inhibits lung cancer cell migration and invasion in vitro

**Figure 2:**
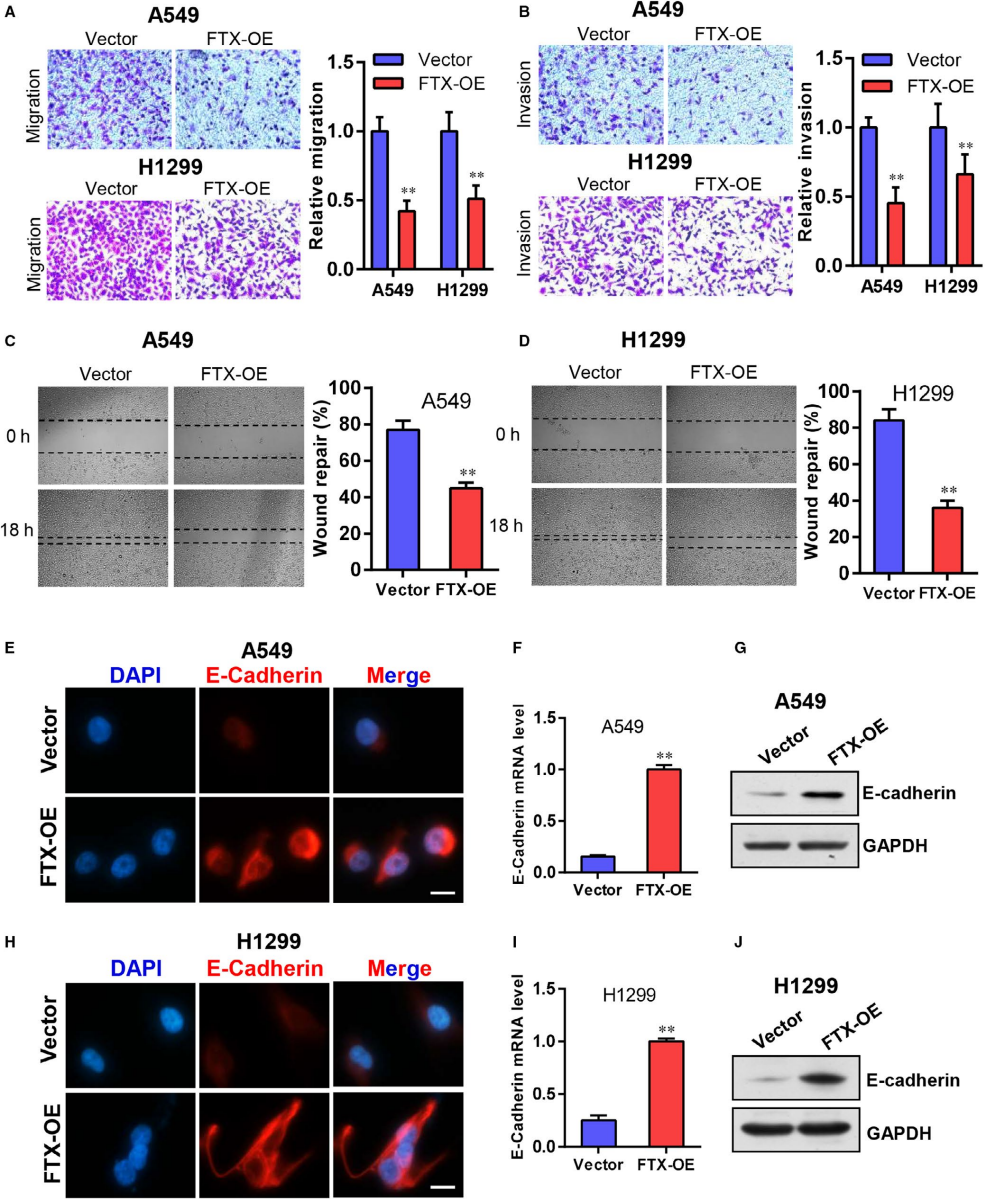
FTX inhibits lung cancer cell migration and invasion in vitro. A, B, Transwell representative images of migration (A) and invasion (B) of vector and FTX‐OE A549 and H1299 cells. Statistic results were shown on the right. Values were shown as mean ± SD from three independent experiments. **P < .01 compared with vector control cells. C, D, Wound healing representative images of vector and FTX‐OE A549 (C) and H1299 (D) cells. Statistic results were shown on the right. Values were shown as mean ± SD from three independent experiments. **P < .01 compared with vector control cells. E, Representative images of immunofluorescence staining of E‐cadherin in vector and FTX‐OE A549 cells. Cell nuclei were stained by DAPI. Scale bar 20 µm. F, Real‐time PCR analysis of E‐cadherin mRNA levels of vector and FTX‐OE A549 cells normalized to GAPDH. Values were shown as mean ± SD from three independent experiments. **P < .01 compared with vector control. G, E‐cadherin protein levels of vector and FTX‐OE A549 cells. GAPDH was used as a loading control. H, Representative images of immunofluorescence staining of E‐cadherin in vector and FTX‐OE H1299 cells. Cell nuclei were stained by DAPI. Scale bar 20 µm. I, Real‐time PCR analysis of E‐cadherin mRNA levels of vector and FTX‐OE H1299 cells normalized to GAPDH. Values were shown as mean ± SD from three independent experiments. **P < .01 compared with vector control. J, E‐cadherin protein levels of vector and FTX‐OE H1299 cells. GAPDH was used as a loading control

Firstly, we investigated whether forced expression of FTX had an effect on migratory and invasive capabilities of lung cancer cells. Results of transwell assay showed that FTX overexpression significantly inhibited cell migration and invasion of A549 and H1299 cells (Figure 2A,B). In addition, wound healing assay also showed that FTX‐OE A549 and H1299 cells migrated more slowly in comparison with vector control cells (Figure 2C,D). E‐cadherin is an important cell adhesion molecule that modulates cell migration and invasion. The result of immunofluorescence staining assay suggested that E‐cadherin expression was significantly increased in FTX overexpressed A549 cells compared with control cells (Figure 2E). Real‐time PCR assay and Western blot assay also confirmed indicated changes in E‐cadherin expression (Figure 2F,G). Same experiments were also performed in H1299 cells, and similar results were acquired (Figure 2H‐J). These data suggest that FTX is involved in regulating lung cancer migration and invasion in vitro.

### 3.3 FTX inhibits lung cancer proliferation in vitro

**Figure 3:**
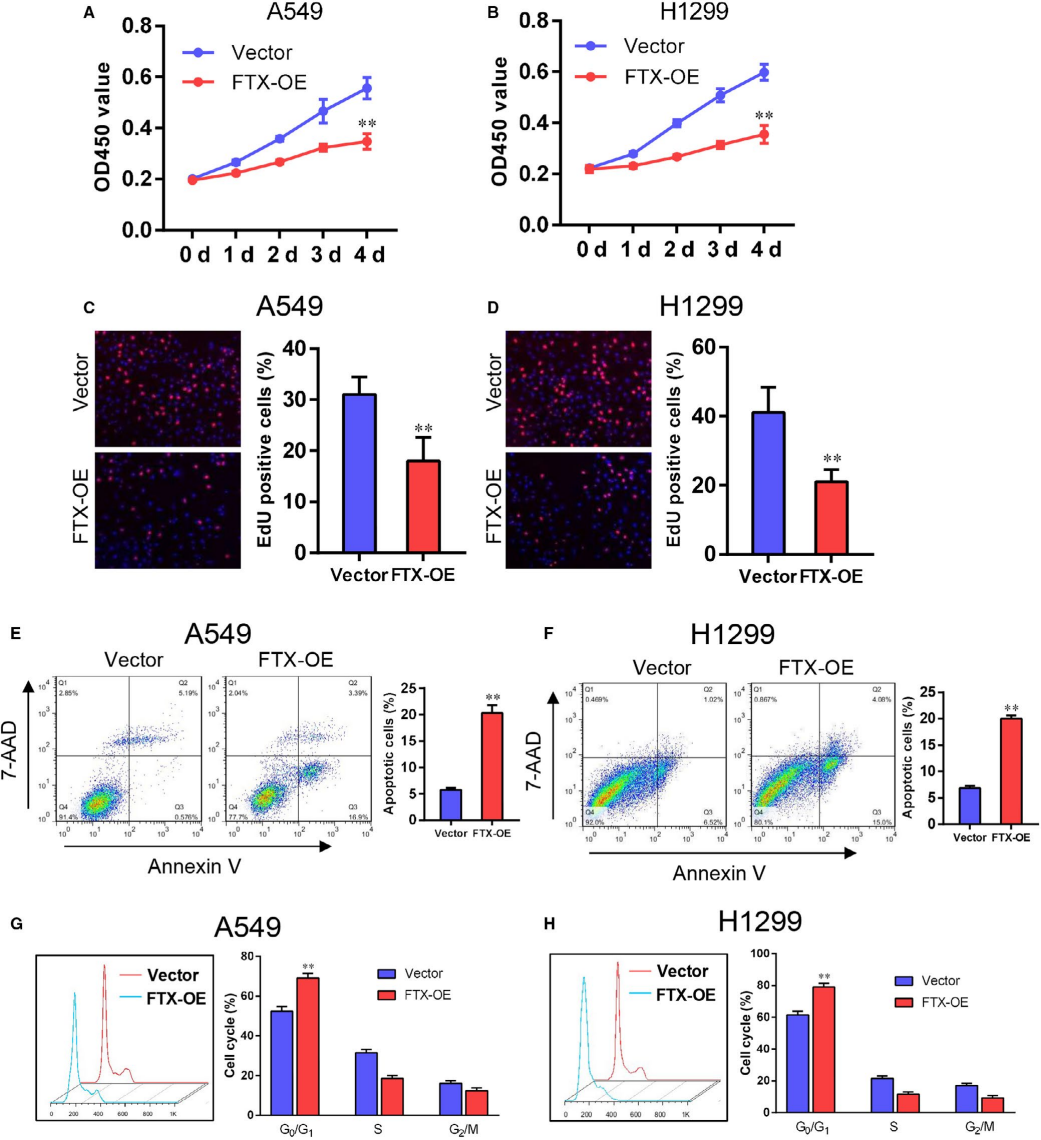
FTX inhibits lung cancer proliferation in vitro. A, B, Growth curves of vector and FTX‐OE A549 (A) and H1299 (B) cells. Values at indicated time‐points were shown as mean ± SD from five independent tests. C, D, EdU proliferation analysis of vector and FTX‐OE A549 (C) and H1299 (D) cells. Representative images of Edu‐positive cells were shown on the left. Statistic data were shown on the right. Values were shown as mean ± SD from three independent experiments. **P < .01 compared with vector control cells. E, F, Apoptosis analysis of vector and FTX‐OE A549 (E) and H1299 (F) cells. Cell flow cytometry data were shown on the left. Statistic data were shown on the right. Values were shown as mean ± SD from three independent experiments. **P < .01 compared with vector control cells. G, H, Cell cycle analysis of vector and FTX‐OE A549 (G) and H1299 (H) cells. Cell flow cytometry data were shown on the left. Statistic data were shown on the right. Values were shown as mean ± SD from three independent experiments. **P < .01 compared with vector control cells

We next explored the impact of FTX overexpression on lung cancer cell growth. The result of CCK‐8 assay suggested that FTX overexpression significantly suppressed A549 and H1299 cell proliferation (Figure 3A,B). Similarly, EdU cell proliferation assay suggested that FTX overexpression inhibited cell proliferation in these two cell lines (Figure 3C,D). Furthermore, cell apoptosis assay and flow cytometry analysis suggested that up‐regulated FTX expression promoted apoptosis of A549 and H1299 cells (Figure 3E,F). Cell cycle analysis suggested that FTX overexpressed A549 and H1299 cells were undergoing cell cycle arrest at G_0_/G_1_ phase (Figure 3G,H). These results above suggest that FTX suppresses lung cancer cell proliferation in vitro.

### 3.4 FTX inhibits lung cancer cell metastasis in vivo

**Figure 4:**
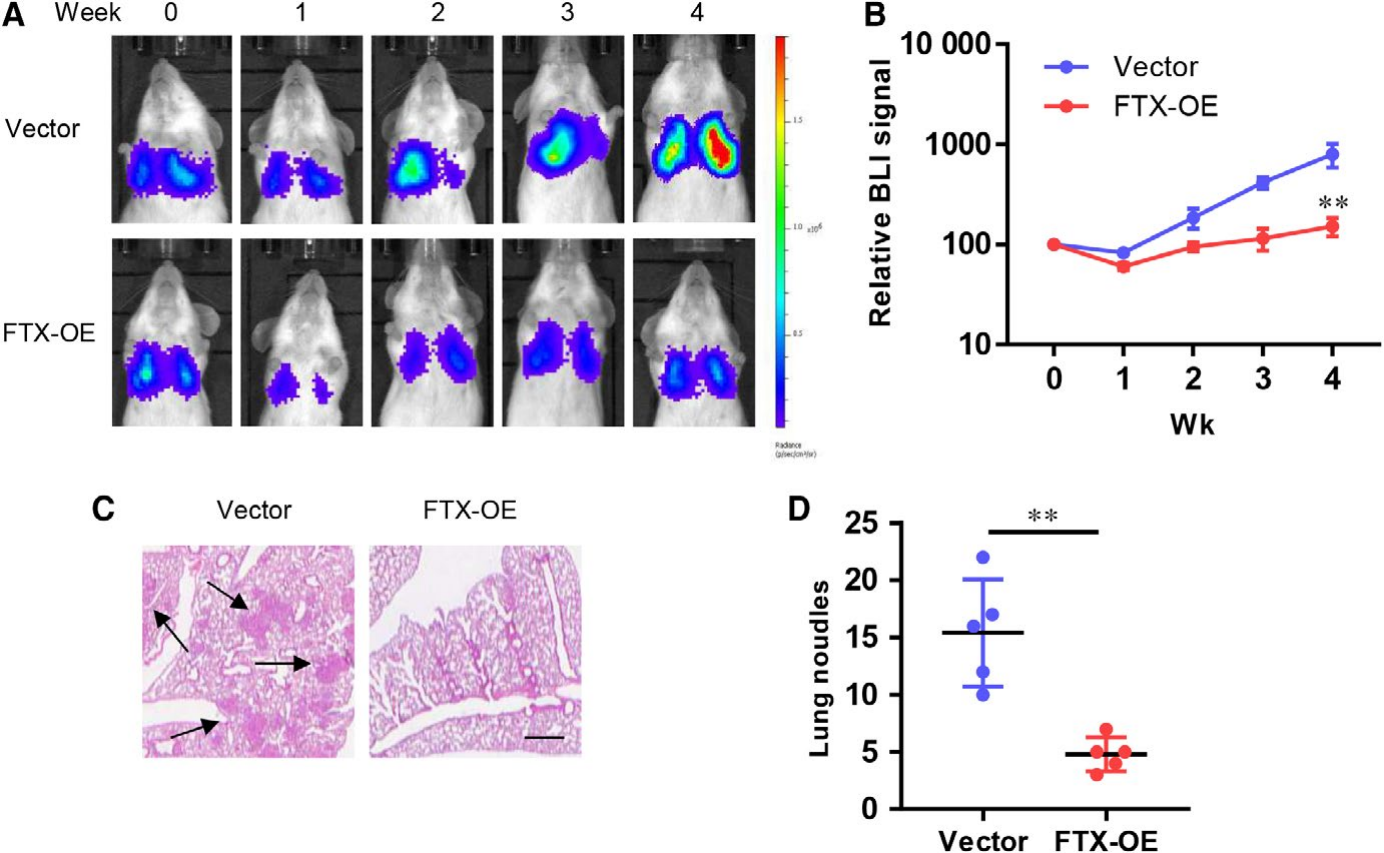
FTX inhibits lung cancer cell metastasis in vivo. A, Representative bioluminescent images of SCID mice injected with control or FTX‐OE A549 cells at indicated time. B, Statistics of normalized bioluminescent signal of SCID mice injected with control or FTX‐OE A549 cells at indicated time. **P < .01 compared with vector control group. C, Representative images of H&E staining of lungs from sacrificed SCID mice. Scale bar = 300 µm. D, Statistic result of lung nodules from SCID mice injected with control or FTX‐OE A549 cells (n = 5). **P < .01 compared with vector control group

To explore the function of FTX in lung cancer metastasis in an in vivo mouse model, we injected vector control or FTX‐OE A549 bioluminescent cells into severe combined immunodeficiency (SCID) mice through tail vein. Bioluminescent imaging system was used to visualize and analyse tumour growth status every week (Figure 4A). Mice injected with FTX‐OE A549 cells showed significantly reduced bioluminescent signals compared with control A549 cells (Figure 4B). Consistently, the number of lung tumour nodules was significantly decreased in mice injected with FTX‐OE A549 cells compared with control A549 cells (Figure 4C,D). These results indicate that FTX can inhibit lung cancer metastasis in vivo.

### 3.5 FTX inhibits lung cancer proliferation in vivo

**Figure 5:**
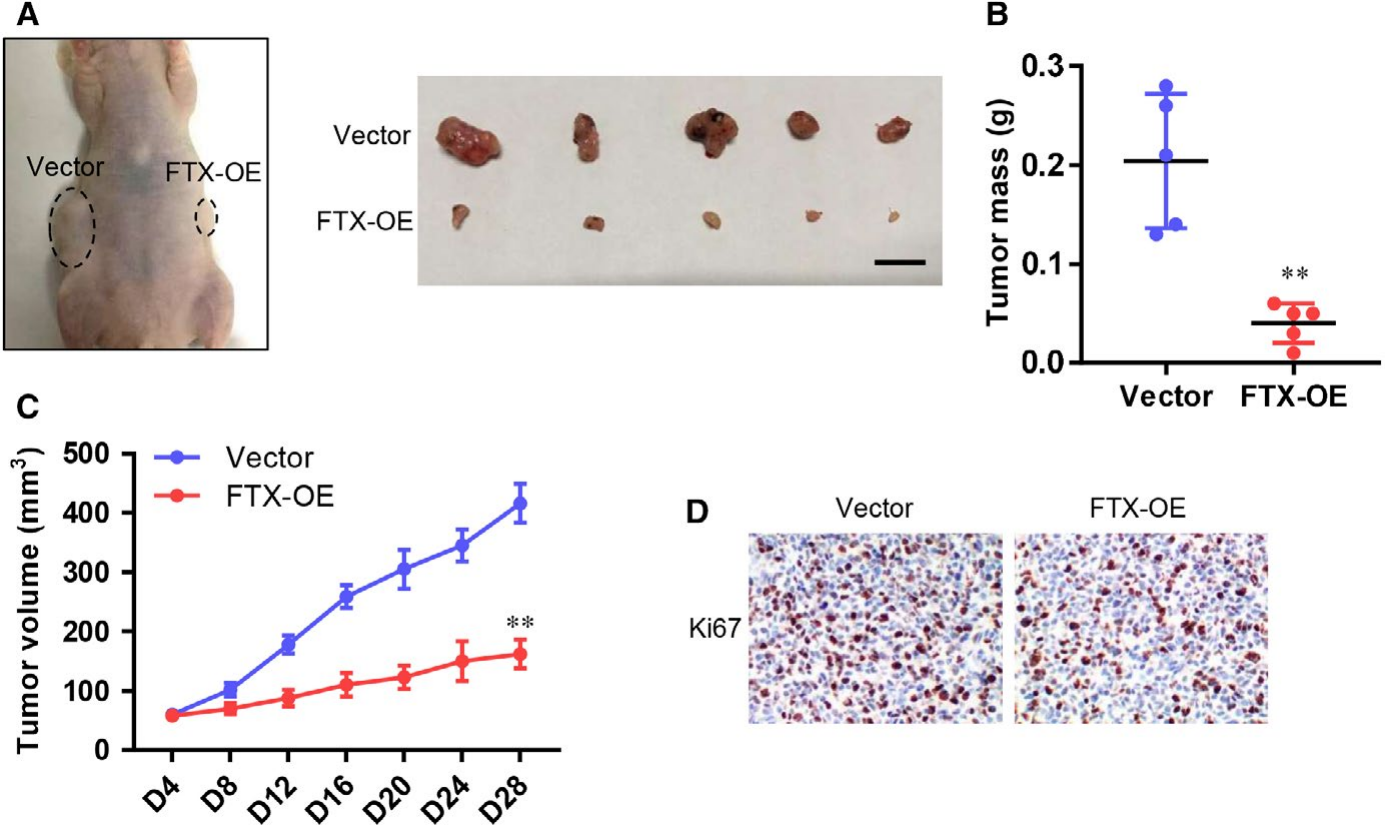
FTX inhibits lung cancer proliferation in vivo. A, Tumour tissues of vector and FTX‐OE A549 cells in nude mice (n = 5). Scale bar = 1cm. B, Weight of excised tumours from nude mice of vector and FTX‐OE groups. C, Tumour volume curves of tumours derived from vector and FTX‐OE A549 cells. Values at indicated time‐points were shown as mean ± SD. **P < .01 compared with vector control. D, Immunohistochemical staining of Ki‐67 in histological sections of tumours form nude mice of vector and FTX‐OE groups. Scale bar 100µm

Then, we used a xenograft mouse model to clarify that FTX had growth‐inhibiting effect on lung cancer cells. We subcutaneously injected vector control and FTX‐OE A549 cells into different flanks of nude mice. In a same mouse, tumour derived from FTX‐OE A549 cells developed more slowly than the one from control A549 cells (Figure 5A). Tumour mass and volume of FTX‐OE group were remarkably reduced in contrast with control group (Figure 5B,C). Moreover, the expression of Ki‐67, a marker of cell proliferation, was significantly decreased in tumours of FTX‐OE group (Figure 5D). These in vivo data suggest FTX inhibits lung cancer proliferation.

### 3.6 FTX sponges miR‐200a‐3p to activate FOXA2 expression in lung cancer cells

**Figure 6:**
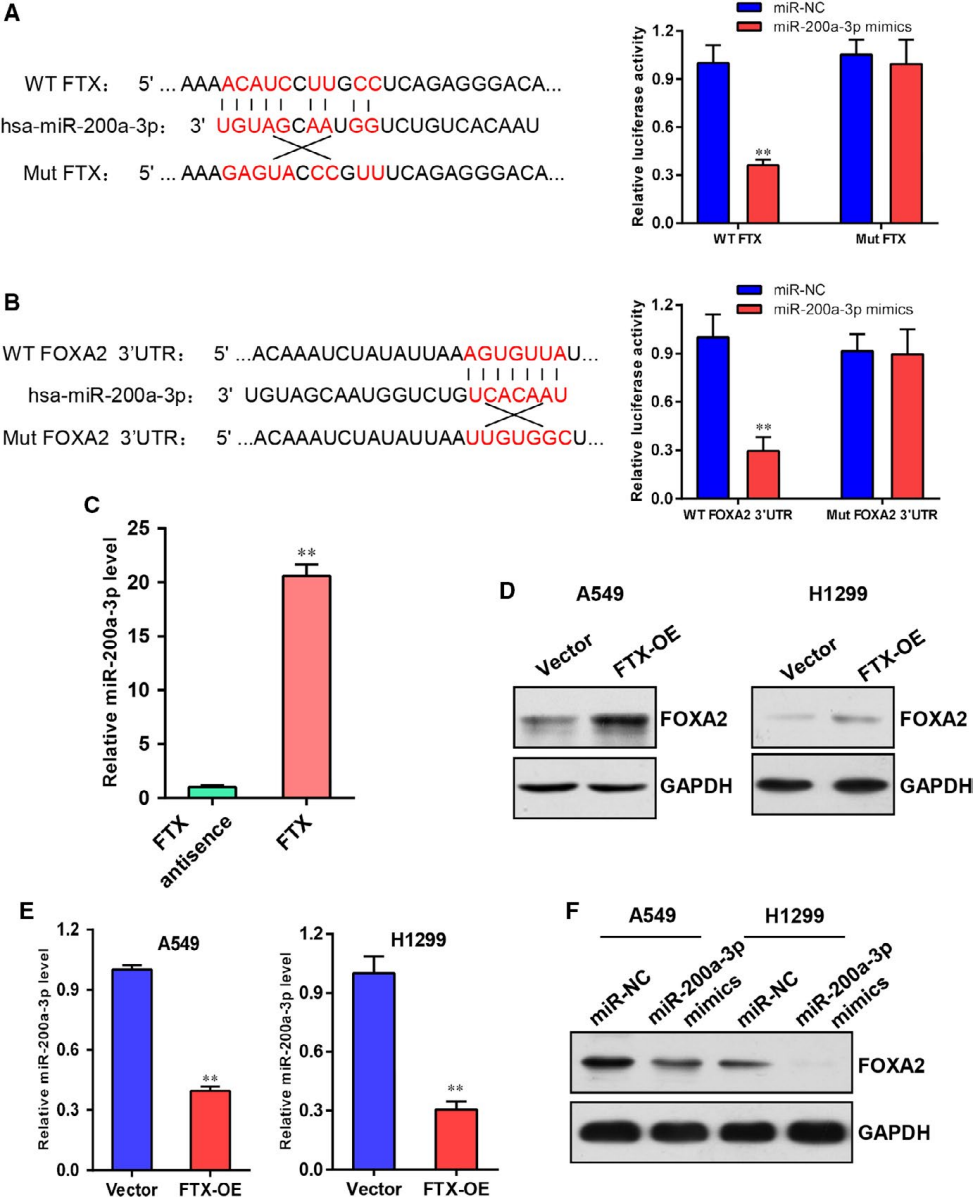
FTX sponges miR‐200a‐3p to activate FOXA2 expression in lung cancer cells. A, B, Wild‐type and mutated miR‐200a‐3p binding sites on FTX and FOXA2 3’UTR region (left). Luciferase reporter gene analysis of A549 cells transfected with miR‐NC, miR‐200a‐3p mimics and wild‐type or mutated vectors (right). **P < .01 compared with miR‐NC. C, Real‐time PCR analysis of coprecipitated miR‐200a‐3p by biotin‐labelled FTX. FTX antisense was used as negative control. **P < .01 compared with FTX antisense group. D, Western blot analysis of FOXA2 expression in vector and FTX‐OE A549 and H1299 cells. GAPDH was used as a loading control. E, Real‐time PCR analysis of miR‐200a‐3p mRNA levels in vector and FTX‐OE A549 and H1299 cells normalized to U6. **P < .01 compared with vector control. F, Western blot analysis of FOXA2 protein levels in A549 and H1299 cells transfected with miR‐NC or miR‐200a‐3p mimics. GAPDH was used as a loading control

To explore potential molecular mechanism of FTX in lung cancer, we predicted competing endogenous (ceRNA) regulatory networks of FTX using online bioinformatics programmes including Targetscan, DIANA, starBase and RNAhybrid. We found that FTX might interact with miR‐200a‐3p to regulate the expression of transcription factor FOXA2. Then, we examined this hypothesis using Luciferase Reporter Gene Assay. We constructed wild‐type or mutated miR‐200a‐3p binding sites in FTX gene and FOXA2 3’UTR region into luciferase reporter gene vectors and then transfected these vectors and miR‐200a‐3p mimics or miR‐NC into A549 cells. The results indicated that measurements of luciferase activity in wild‐type vectors were remarkably suppressed by miR‐200a‐3p mimics compared with miR‐NC, while luciferase activity of mutant vectors was not affected by miR‐200a‐3p mimics (Figure 6A,B). RNA pull‐down assay was used to further confirm the interaction between FTX and miR‐200a‐3p, and the result suggested that miR‐200a‐3p interacted with FTX but not FTX antisense (Figure 6C). In addition, we checked FOXA2 protein levels in A549 cells and H1299 cells and found that FOXA2 expression was increased in FTX overexpressed cells compared with control cells (Figure 6D). Real‐time PCR analysis showed that miR‐200a‐3p mRNA levels were conversely reduced in FTX overexpressed A549 and H1299 cells in comparison with control cells (Figure 6E). Moreover, transfection with miR‐200a‐3p mimics into A549 and H1299 cells resulted in down‐regulation of FOXA2 protein levels (Figure 6F). These collecting data demonstrate that FTX acts as a sponge of miR‐200a‐3p to activate FOXA2 expression in lung cancer cells.

### 3.7 The function of FTX in lung cancer cells depends on activation of FOXA2 expression

**Figure 7:**
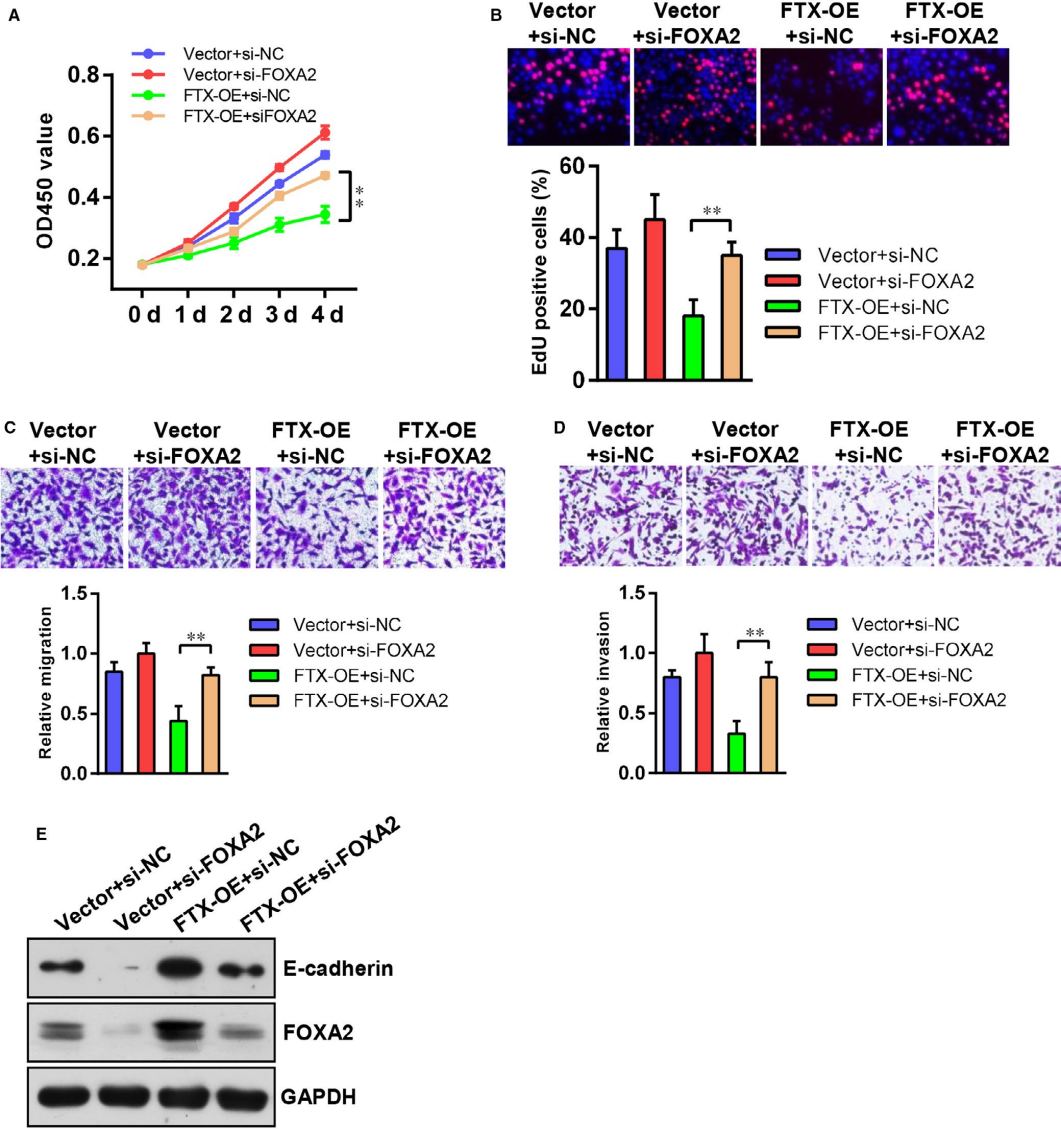
The function of FTX in lung cancer cells depends on activation of FOXA2 expression. A, Growth curves of vector + si‐NC, vector + si‐FOXA2, FTX‐OE + si‐NC and FTX‐OE + siFOXA2 A549 cells. Values at indicated time‐points were shown as mean ± SD from five independent tests. B, EdU proliferation analysis of vector + si‐NC, vector + si‐FOXA2, FTX‐OE + si‐NC and FTX‐OE + siFOXA2 A549 cells. Representative images of Edu‐positive cells were shown at the top. Statistic data were shown at the bottom. Values were shown as mean ± SD from three independent experiments. **P < .01 compared with vector control cells. C, D, Transwell representative images of migration (C) and invasion (D) of vector + si‐NC, vector + si‐FOXA2, FTX‐OE + si‐NC and FTX‐OE + siFOXA2 A549 cells. Statistic results were shown at the bottom. Values were shown as mean ± SD from three independent experiments. **P < .01 compared with vector control cells. E, Western blot analysis of E‐cadherin and FOXA2 protein levels in vector + si‐NC, vector + si‐FOXA2, FTX‐OE + si‐NC and FTX‐OE + siFOXA2 A549 cells. GAPDH was used as a loading control

FOXA2 is a transcriptional regulator which has a critical impact on lung cancer cell growth and metastasis. To test whether FOXA2 is critical for FTX‐mediated inhibition of lung cancer cell growth and metastasis, we silenced FOXA2 expression in FTX‐OE A549 cells to do rescue experiments. We found that transfection of FOXA2 siRNA partially abrogated FTX overexpression‐mediated suppression of cell proliferation in A549 cells (Figure 7A,B). Also, down‐regulation of FOXA2 was able to restore the migratory and invasive capabilities of A549 cells in vitro (Figure 7C,D), which was accompanied by inhibition of E‐cadherin expression (Figure 7E). These data suggest that the effect of FTX in lung cancer depends on FOXA2.

## 4 DISCUSSION

Emerging researches have reported that long non‐coding RNAs are broadly involved in regulating human physiological and pathological processes. FTX was reported to be involved in Xist gene regulation and X chromosome inactivation. However, the functional role of FTX in lung cancer progression was not clear. In our study, we verified that FTX expression was decreased in NSCLC tissue samples and cell lines. Enforced expression of FTX in lung cancer cells suppressed cancer growth and metastasis in vitro and in vivo. Furthermore, we revealed a novel FTX/miR‐200a‐3p/FOXA2 signalling axis in lung cancer progression.

E‐cadherin is considered to be a major epithelial cell‐cell adhesion molecule that inhibits cell migration and invasion to extracellular matrix.^12^, ^13^ Here, our results revealed that E‐cadherin expression was significantly up‐regulated in FTX overexpressed lung cancer cells, which indicated that FTX inhibited lung cancer cell migration and invasion. Moreover, we found that FTX overexpression in lung cancer cells promoted cell apoptosis and induced cell cycle arrest at G_0_/G_1_ phase. The function of FTX in carcinogenesis is still controversial. It was reported that FTX was up‐regulated in renal cancer and promoted cell proliferation, migration and invasion.^14^ Meanwhile, FTX was reported to promote colorectal cancer progression via sponging miR‐215 and inhibiting phosphorylation of vimentin.^15^ However, FTX was found to inhibit growth and metastasis of hepatocellular carcinoma cells through binding MCM2 and miR‐374a.^9^ In this present study, our findings of FTX in cell growth and metastasis regulation, combined with the expression profile of FTX in lung cancer, indicate that FTX is a potential tumour suppressor gene in lung cancer progression.

The ceRNA hypothesis is wildly used to elucidate the molecular mechanism of lncRNA in gene regulation and has been proved by hundreds of studies.^16^, ^17^, ^18^ We analysed the potential ceRNA network of FTX and validated the hypothesis that FTX sponged miR‐200a‐3p to activate FOXA2 expression in lung cancer cells. The result of RNA pull‐down assay suggested that FTX interacted with miR‐200a‐3p in lung cancer cells, and Luciferase Reporter Gene Assay indicated that miR‐200a‐3p could both target FTX and FOXA2 genes. FTX overexpression activated FOXA2 expression, while transfection of miR‐200a‐3p mimics inhibited FOXA2 expression in lung cancer cells. These results reveal the presence of FTX/miR‐200a‐3p/FOXA2 axis in lung cancer cells. Moreover, FTX overexpression also inhibited miR‐200a‐3p expression in lung cancer cells. Similar phenomena have been reported in many studies on ceRNA regulatory signalling. Markus Stoffel and colleagues reported that lncRNA could trigger miRNA degradation through interacting with it in ceRNA regulation.^19^ Another study reported that lncRNA CCAT2 could interact with miR‐145 to prevent its maturation in colon cancer cells.^20^ Here, the mechanism of FTX‐mediated miR‐200a‐3p decay is still unclear and needs further study. A previous study reported that miR‐200a‐3p was up‐regulated in A549 cells and promoted cell migration,^21^ which is consistent with our findings. FOXA2 is a crucial transcription factor in embryo development. Recently, increasing number of literatures have proved FOXA2 plays a critical role in cancer progression. It was reported that FOXA2 expression levels were decreased in almost all lung cancer cell lines, and FOXA2 acted as a tumour suppressor of lung cancer metastasis by inhibiting Slug gene expression and subsequent EMT process.^22^, ^23^ Meanwhile, Shandy Shahabi et al found that hypermethylation in FOXA2 gene promoter region suppressed FOXA2 expression in LUAD, and ectopic expression of FOXA2 in LUAD cells inhibited cell migration and proliferation through activating LINC00261 expression.^24^ However, FOXA2 was reported to promote proliferation and migration in colon cancer.^25^ Although there are conflicting data of the function of FOXA2 in cancer progression, in the present study, our results suggested that FOXA2 silencing promoted cell proliferation and migration in lung cancer cells. Furthermore, we demonstrated that FOXA2 was a downstream target of FTX, while rescue experiments indicated that silencing of FOXA2 attenuated the effect of FTX on lung cancer growth and metastasis. These data suggest that the function of FTX in lung cancer progression, at least partially, depends on activation of FOXA2 expression.

In summary, our study demonstrates that FTX may function as a tumour suppressor gene and regulate lung cancer development through acting as a sponge of miR‐200a‐3p to promote FOXA2 expression. We reveal a novel role of FTX in cancer cell growth and metastasis, providing a potential biomarker and therapeutic target in lung cancer diagnosis and treatment.

## CONFLICTS OF INTEREST

The authors declare that they have no conflicts of competing interests.

## AUTHOR CONTRIBUTIONS

SJ, JH, YZ and DW planned and carried out the experiments. JH analysed the data. SJ wrote the manuscript with support from JH, YZ and DW. JL collected the clinical samples. WG supervised the project.

## Data Availability

All data generated or analysed during this study are included in this manuscript.
